# Functional characterization of SOX2 as an anticancer target

**DOI:** 10.1038/s41392-020-00242-3

**Published:** 2020-07-29

**Authors:** Shizhen Zhang, Xiufang Xiong, Yi Sun

**Affiliations:** The Cancer Institute of the Second Affiliated Hospital and Institute of Translational Medicine, Zhejiang University School of Medicine, Hangzhou, 310029 China

**Keywords:** Cancer, Cell biology, Cancer, Cancer stem cells

## Abstract

SOX2 is a well-characterized pluripotent factor that is essential for stem cell self-renewal, reprogramming, and homeostasis. The cellular levels of SOX2 are precisely regulated by a complicated network at the levels of transcription, post-transcription, and post-translation. In many types of human cancer, SOX2 is dysregulated due to gene amplification and protein overexpression. SOX2 overexpression is associated with poor survival of cancer patients. Mechanistically, SOX2 promotes proliferation, survival, invasion/metastasis, cancer stemness, and drug resistance. SOX2 is, therefore, an attractive anticancer target. However, little progress has been made in the efforts to discover SOX2 inhibitors, largely due to undruggable nature of SOX2 as a transcription factor. In this review, we first briefly introduced SOX2 as a transcription factor, its domain structure, normal physiological functions, and its involvement in human cancers. We next discussed its role in embryonic development and stem cell-renewal. We then mainly focused on three aspects of SOX2: (a) the regulatory mechanisms of SOX2, including how SOX2 level is regulated, and how SOX2 cross-talks with multiple signaling pathways to control growth and survival; (b) the role of SOX2 in tumorigenesis and drug resistance; and (c) current drug discovery efforts on targeting SOX2, and the future perspectives to discover specific SOX2 inhibitors for effective cancer therapy.

## Introduction

**Fig. 1:**
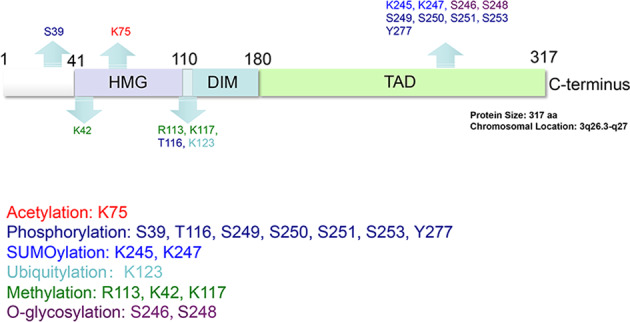
The SOX2 domain structures and the posttranslational modification sites. SOX2 protein consists of 317 amino acids with three functional domains: high mobility group (HMG) domain at the N-terminus, dimerization (DIM) domain at the center, and transactivation (TAD) domain at the C-terminus. SOX2 is subjected to modification at the posttranslational level by acetylation, phosphorylation, SUMOylation, ubiquitylation, methylation, O-Glycosylation, and PARPylation. Note that the PARPylation site has not been identified

The gene of *sex-determining region Y-box 2* (*SOX2*) is located on chromosome 3p26.3-q27, and encodes a protein of 317 amino acids comprising of three main domains: high mobility group (HMG) domain at the N-terminus, dimerization (DIM) domain at the center, and transactivation (TAD) domain at the C-terminus^1^ (Fig. 1). As a transcription factor, SOX2 recognizes and binds to the promoter of various target genes via its TAD domain to trans-activate or -repress their expression, thus regulating various physiological processes.^2^ SOX2 plays a pivotal role in maintenance of the stem cell phenotype of embryonic stem cells (ESCs) during the embryogenesis. *SOX2* deletion in zygotes triggers differentiation of ESCs into trophectoderm (TE)-like cells, leading to failure in embryoblast formation and early embryonic lethality.^3^ The most attractive feature of SOX2 is being one of the Yamanaka factors, whose ectopic expression along with Oct4, Klf4, and c-Myc converts mouse embryonic fibroblasts into induced pluripotent stem cells (iPSCs).^4^ Following the discovery of the key roles of SOX2 in ESCs and iPSCs, SOX2 expression in human cancers has been widely investigated. The SOX2 amplification or overexpression was found in at least 25 different human cancers, and forced SOX2 expression promotes neoplastic progression by accelerating cancer cell proliferation, migration, invasion, and metastasis.^5^ Moreover, elevated SOX2 expression is positively correlated with drug resistance and poor survival of cancer patients.^5,6^ Therefore, targeting SOX2 appears to be a very attractive therapeutic avenue for cancer treatment.^7^

## Role in regulation of embryonic development and stem cell self-renewal

The first lineage specification event in mammalian embryo is the differentiation of blastocysts into inner cell mass (ICM) and TE.^8^ SOX2 is initially expressed in random cells at morula stages (2.5 days postcoitum), and later restrictedly in ICM at blastocyst stages (3.5 days postcoitum).^3^ SOX2 is therefore considered as an earliest marker of ICM formation.^9^ Importantly, zygotic deletion of *SOX2* causes the failure in the formation of the pluripotent epiblast, but without affecting the TE formation, and early embryonic lethality.^3^ While maternal SOX2 protein expression persists in preimplantation embryos,^9^ and depletion of both maternal or embryonic *SOX2* via RNAi disrupts the formation of TE or cavity and results in an early arrest of embryos at the morula stage, indicating that SOX2 is essential for the segregation of the ICM and TE.^9^ Consistently, *SOX2* deletion in embryos fails to support the derivation of ESCs from the ICM,^3^ whereas *SOX2* deletion in the already established ESCs still leads to inappropriate differentiation into TE-like cells.^10^ SOX2 is, therefore, critical for the self-renewal and differentiation of ESCs. The subsequent studies indicate that SOX2 cooperates with other dosage-sensitive transcription factors, such as Oct4 and Nanog, to maintain self-renewal state and repress differentiation of ESCs by efficiently binding to the promoter/enhancer regions and affecting target genes activation.^11–13^

**Fig. 2:**
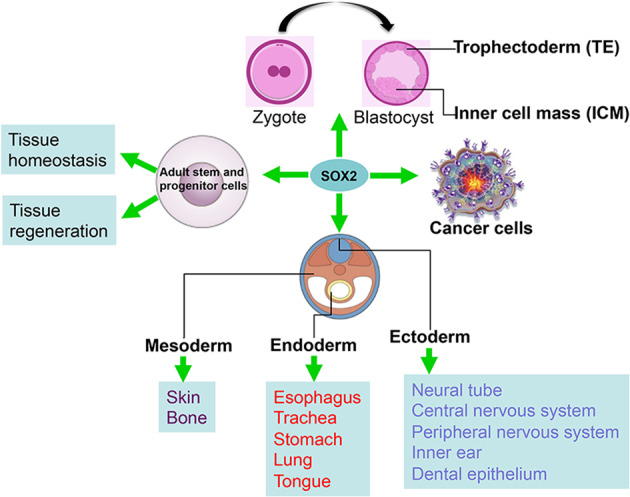
The role of SOX2 in embryonic development, pluripotency, and homeostasis. SOX2 is initially expressed in pluripotent founder cells of blastocyst, and plays the key role in the embryonic development of three cell linages of ectoderm, endoderm, and mesoderm. SOX2 expression in adult stem and progenitor cells is essential for tissue homeostasis and regeneration. Dysregulation of SOX2 contributes to tumorigenesis

Moreover, SOX2 plays an important role in the development of three germ layers: the endoderm, ectoderm, and mesoderm (Fig. 2). For the ectodermal lineage, SOX2 is directly involved in the development of central nervous system (CNS) and peripheral nervous system by regulating the proliferation and differentiation of fetal progeny cells.^14,15^ The *SOX2* depletion results in cell-cycle exit and differentiation of CNS progenitors.^16^ SOX2 activity is also critical for the differentiation of retinal progenitor cells via regulating the NOTCH1 signaling pathway.^17^ In addition, SOX2 plays an important role in the differentiation of subsets of neurons. For example, SOX2 mutant neural stem cells exhibit morphologically immature β-tubulin-positive neuronal-like cells, and *SOX2* neural knockout mice manifest diminished GABAergic interneurons in newborn cortex and in adult olfactory bulb.^18,19^ SOX2 also serves as an early permissive factor in the development of other ectoderm-derived tissues, including the sensory cells within cochlea and dental epitheliums.^20,21^ For endoderm development, SOX2 plays a dose-dependent role in organ specification of the foregut. For example, the anterior part of the foregut with high SOX2 expression differentiates into esophagus and forestomach, while the low SOX2 expression gives rise to trachea and posterior stomach.^22^ The differentiation of primary lung bud into mature lung and the morphogenesis of the embryonic tongue into taste sensory cells are also regulated by SOX2 in a dose-dependent manner.^23,24^ Similarly, SOX2 is involved in mesoderm development, such as skin and osteoblast development.^25^ SOX2 is regarded as a biomarker of dermal stem cell, since SOX2^+^ dermal cells are originated from self-renewing skin-derived precursor cells.^25^ And SOX2^+^ dermal cells are able to induce hair morphogenesis and differentiate into multiple dermal cell types upon transplanting into nude mice.^26^ In addition, *SOX2* overexpression in osteoblast cell prevents its differentiation, whereas *SOX2* knockout induces a senescence-like phenotype.^27^ Consistently, the in vivo mouse experiments with osteoblast-specific *SOX2* knockout confirm that SOX2 is essential for osteoblast self-renewal,^28^ while *SOX2* transgenic expression impairs mature osteoblast function.^29^ Taken together, SOX2 plays an essential role in embryonic development and stem cell self-renewal.

## Role in regulation of key signaling pathways

**Fig. 3:**
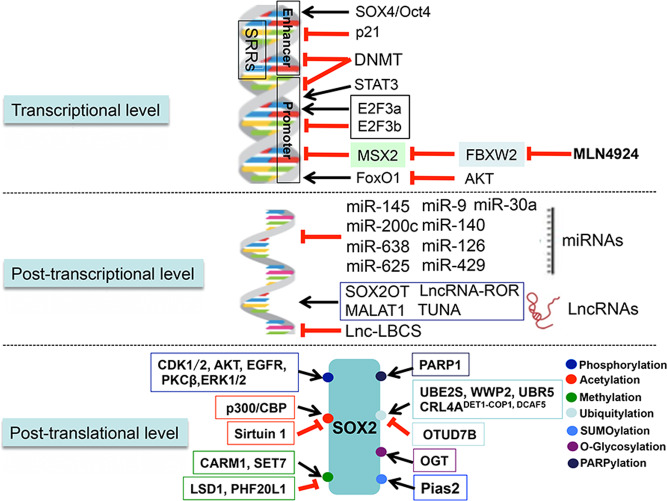
SOX2 is regulated at multiple levels. The SOX2 levels and activities are precisely regulated by a complicate network at the levels of transcription, post-transcription, and post-translation, as shown. At the transcriptional levels, SOX2 mRNA is either up- or downregulated by its enhancers, and other transcription activators or repressors. The post-transcriptional regulators are mainly the miRNAs and lncRNAs, that control the stability of SOX2 mRNA. The posttranslational regulators are a variety of enzymes that modify specific residues on SOX2 protein, leading to alterations in SOX2 activity, subcellular localization, and stability. MALAT1 metastasis-associated lung adenocarcinoma transcript 1, TUNA Tcl1 upstream neuron-associated lncRNA, DNMT DNA methyltransferase, OTUD7B OTU domain-containing protein 7B, OGT O-GlcNAc transferase

**Fig. 4:**
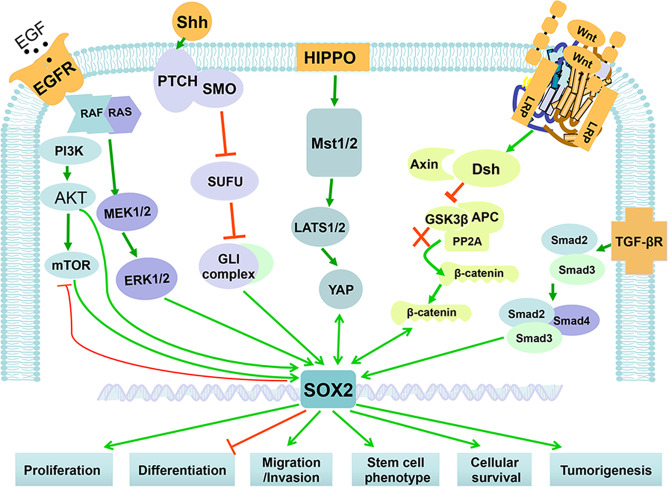
SOX2 cross-talks with various signaling pathways. SOX2 cross-talks with a variety of signaling pathways that regulate proliferation, survival, and tumorigenesis, including EGFR, SHH, HIPPO, WNT/β-Catenin, and TGF-β/Smads signaling pathways. In general, SOX2 positively or negatively regulates proliferative or antiproliferative signaling pathways, respectively, leading to enhanced proliferation, survival, and tumorigenesis

As a critical transcriptional factor performing significant biological functions, the levels of SOX2 need to be precisely regulated. Indeed, SOX2 mRNA and protein are subjected to regulations at the transcriptional, posttranscriptional, and posttranslational levels (Fig. 3). Finely tuned SOX2 then regulates multiple signal transduction pathways involved in numerous physiological processes, as well as pathological processes, if dysregulated (Fig. 4).^30,31^

### SOX2 is regulated at multi-levels

#### At the transcriptional level

The mammalian *SOX2* gene is localized within a gene desert, being in a large genomic region mostly devoid of other protein coding genes. There are at least 27 distinct enhancers that can be transcriptionally activated to induce SOX2 during neurosensory development by the analysis of a 200 kb chicken gene region surrounding the *SOX2* single exon. Importantly, most of these enhancers are also located in conserved regions in mammals.^32^ Thus, it is not surprising that the mammalian *SOX2* gene is transcriptionally regulated by several distinct distal enhancers during different stages of development. However, only few enhancers have been identified as functionally active in mammalian cells,^33^ and further studies are needed to clearly define the regulatory enhancers of the *SOX2* gene that are active in specific cellular situations.

Two early identified *SOX2* enhancers, SOX regulatory regions (*SRR*) 1 and 2, are capable of influencing the activity of promoter of *SOX2* and specifically exert their functions when cells are in an undifferentiated state.^34^*SRR1* displays activity in the promoter constructs expressed in ESCs, but deletion of *SRR1* has minimal impact on SOX2 pluripotency.^35^ However, deletion of *SRR1* containing region in ~5.7–~3.3 kb upstream of the *SOX2* coding region abolishes the SOX2 expression in telencephalic neural stem cells and precursors during murine development.^36,37^*SRR2* is another enhancer located at ~2.5 kb downstream of the *SOX2* coding region. By activating the SOX2 expression, *SRR2* is not only active in mouse ESCs, but also serves as a biomarker for isolation of human iPSCs.^38^ In addition, other SRR regions located in the downstream distal enhancers, such as *SRR18*, *SRR107*, and *SRR111*, appear to interact with the proximal enhancers to form a large chromatin loop to enhance SOX2 transcription in ESCs.^35^ Interestingly, ectopic overexpression of SOX2 in ESCs inhibits the endogenous SOX2 expression to trigger cell differentiation, suggesting that SOX2 could control its own expression via a negative feedback loop.^39,40^ Some other SOX family members are also likely to positively regulate SOX2 expression. For example, SOX4 in cooperation with Oct4 forms a complex at the enhancer of *SOX2* to enhance its expression, therefore maintaining the stemness of glioma-initiating cells.^41^ In melanoma cells, epidermal growth factor receptor (EGFR) activation enhances STAT3 nuclear translocation and binding to the *SOX2* promoter, leading to increased SOX2 expression to facilitate cell survival and self-renewal.^42^ In neural stem cells, two isoforms of the cell-cycle regulator E2F transcription factor 3 (E2F3), E2F3a, and E2F3b, control SOX2 transcription by targeting *SOX2* locus either positively or negatively^43^ (Fig. 3).

Furthermore, SOX2 expression is also subjected to negative regulation at the transcriptional level. For example, the methylation of the *SOX2* promoter by DNA methyltransferase (DNMT) inhibits SOX2 transcription, and this hypermethylation of *SOX2* appears to be a critical epigenetic event, leading to SOX2 silencing in several types of human cancers, including esophageal cancer,^44^ gastric cancer (GC),^45^ and endometrial cancer.^46^ However, the loss of SOX2 in these subgroups of cancers is associated with the worse clinical characteristics and poor prognosis,^44–46^ which is an interesting subject for future investigation. Furthermore, DNMT is involved in the dynamic DNA methylation of SOX2 at the super-enhancers region, which is essential for transcriptional and cellular heterogeneity of ESCs.^47^ Furthermore, in breast cancer, the activated AKT increases the phosphorylation and degradation of its downstream target FoxO1, which inhibits *SOX2* transcription.^48^ The expression of pseudokinase Tribble 3 promotes breast cancer stemness and progression by activating the AKT1–FoxO1–SOX2 axis.^48^ The cyclin-dependent kinase inhibitor p21 is capable of controlling adult neural stem cell expansion by directly binding to the *SOX2* enhancer and negatively regulates *SOX2* transcription.^49^ Recently, a homeobox-containing transcription factor, muscle segment homeobox-2 (MSX2) was found to destabilize the pluripotency circuitry by acting as a transcription repressor to directly bind to the *SOX2* promoter and inhibit *SOX2* transcription, which is essential for mesendoderm differentiation.^50^ Our most recent study showed that SCF^FBXW2^ is the novel E3 ligase that targets MSX2 for ubiquitylation and degradation, consequently activating *SOX2* transcription by removing MSX2 repression, thus inducing stem cell property of breast cancer cells. As such, MLN4924, a small molecule inhibitor of protein neddylation,^51^ significantly downregulated *SOX2* transcription by inactivating SCF^FBXW2^ and subsequently suppressed cancer cell stemness^52^ (Fig. 3). Finally, SOX2 transcription activity is subjected to negative regulation by a SOX2 mutant. SOX2 contains two nuclear localization signals (NLS) that are required for SOX2 nuclear localization and transcription activity.^53^ A SOX2 NLS mutant acts in a dominant negative manner to form a complex with wild-type SOX2 to retain it in the cytoplasm, thus suppressing its transcription activity and inhibiting expression of its downstream targets such as *Fgf4*, *Oct4*, and *Nanog*.^53^

#### At the posttranscriptional level

A growing list of microRNAs (miRNAs) has been identified to regulate SOX2 expression at the posttranscriptional level. Endogenous miR-145 represses *SOX2* expression by directly targeting the 3′-UTR of the *SOX2* mRNA to impair the self-renewal capability of ESCs.^54^ Likewise, miR-200c inhibits the SOX2 expression by directly targeting the conserved binding site of the *SOX2* mRNA at the 3′-UTR site, resulting in dysregulated cell cycle and neuronal differentiation.^55^ In human cancers, loss of miR-638 in hepatocellular carcinoma cells results in increased cell invasive capacity by targeting *SOX2* expression. As such, miR-638 is considered as a tumor suppressor, whose downregulation in hepatocellular carcinoma is associated with worse prognosis of patients.^56^ MiR-625 has been shown to inhibit *SOX2* translation via targeting its 3′-UTR site, thus significantly suppressing esophageal cancer cell growth and migration, which explains why it is expressed at an extremely low level in human esophageal cancer tissues.^57^ Moreover, miR-9, miR-30a, miR-140, miR-145, and miR-126, are all shown to act as tumor suppressors and negatively regulate the SOX2 expression in human cancers.^58–62^ Conversely, several highly expressed oncogenic miRNAs upregulate SOX2 levels. For example, in breast carcinoma specimen, miR-378 expresses at a higher level with a positively correlated high level of SOX2.^63^ Human glioblastoma cells transfected with miR-378 form more and larger spheres and colonies by increasing SOX2 levels via a mechanism of targeting vimentin, rather than direct *SOX2* binding.^63^ The miR-429 is highly expressed in colorectal cancers, which is significantly associated with tumor progression and poor prognosis, and miR-429 overexpression suppresses cell apoptosis via a mechanism of directly targeting SOX2.^64^ In GC cells, miR-126 inhibits SOX2 expression by binding to its 3′-UTR, which unexpectedly contributes to carcinogenesis^65^ (Fig. 3).

Besides miRNA regulation of SOX2, SOX2 itself also regulates a number of miRNAs. A genome-scale location analysis showed that SOX2 is associated with regulatory regions of several miRNA genes in human ESCs.^11^ A ChIP-seq analysis suggested that SOX2 could bind to over 100 miRNAs in LN229 glioblastoma cell.^66^ In breast cancer cells, SOX2 negatively regulates miR-452 expression to decrease the VEGFA-driven SNAI2 expression, thus promoting cell motility and invasive ability.^67^ SOX2 knockdown significantly downregulates miR-181a-5p and miR-30e-5p in breast cancer cells, leading to suppression of cancer cell proliferation, migration, and invasion.^68^ In glioblastoma, SOX2 is able to inhibit miR-296-5p via promoter DNA methylation, thereby regulating stem cell phenotype of glioblastoma.^69^ Interestingly, several miRNAs, such as miR-200c and miR-145, are shown to form double negative feedback loops with SOX2, establishing a cross-talk network.^66,70^

In addition to the SOX2-miRNAs cross-talks, a cross-regulation also exists between SOX2 and the long noncoding RNAs (lncRNAs), a class of nonprotein-coding RNAs, consisting of more than 200 nucleotides. Structurally, SOX2 directly interacts with lncRNAs with high affinity through its HMG DNA-binding domain.^71^ Interestingly, functional *SOX2* gene is embedded in the third intron of a long multi-exon noncoding RNA gene, which is known as *SOX2* overlapping transcript *(SOX2OT)*.^72^*SOX2* and *SOX2OT* are co-expressed in ESCs and both are transcribed in the same orientation.^73^ In breast cancer cells, the ectopic expression of *SOX2OT* significantly promotes *SOX2* expression by almost 20-folds, leading to increased anchorage-independent growth,^74^ whereas conditional knockdown of *SOX2OT* inhibits the *SOX2* transcription to reduce cancer cell viability and ameliorate cell migration and invasion in bladder and cervical cancer cells.^75,76^ In addition, a conserved lncRNA, metastasis-associated lung adenocarcinoma transcript 1 (MALAT1), positively regulates the *SOX2* expression by directly binding to *SOX2* mRNA.^77^ MALAT1 knockdown efficiently suppresses SOX2 expression, whereas MALAT1 overexpression increases SOX2 expression to enhance stemeness of GC and glioma cells.^77,78^ Tcl1 upstream neuron-associated lncRNA (TUNA), a pluripotency essential lncRNA, is shown to activate *SOX2* expression by recruiting three RNA-binding proteins, PTBP1, hnRNP-K, and NCL to the *SOX2* promoter, which is critical for neural differentiation of ESCs and cancer cell stemness.^79,80^ In bladder cancer, lnc-LBCS is markedly downregulated in cancer stem cells (CSCs), whereas its overexpression suppresses the self-renewal of stem cells by inhibiting SOX2 expression via mediating histone H3 lysine 27 tri-methylation.^81^ Finally, miR-145 and lncRNA-ROR recognize the same sequence in the 3′-UTR of *SOX2*, suggesting a potential competitive binding. Indeed, lncRNA-ROR promotes *SOX2* expression via competing off miR-145, eventually modulating the pluripotency of human amniotic epithelial stem cells^82^ (Fig. 3).

#### At the posttranslational level

The posttranslational modification of SOX2 by phosphorylation, SUMOylation, methylation, acetylation, poly(ADP)-ribosylation (PARPylation), O-Glycosylation, and ubiquitylation, is another type of regulatory mechanism that mainly affects the activities of SOX2 (Figs. 1 and 3). Phosphorylation is the most common type of posttranslational modification of SOX2. Several serine and threonine resides on SOX2 are known to be phosphorylated in cultured cells.^83–85^ Specifically, CDK1 appears to phosphorylate SOX2 at S249-S250-S251, which is required for SOX2 nuclear localization and transcriptional activity, thereby promoting survival of melanoma cells.^86^ CDK2 directly phosphorylates SOX2 at S39 and S253, which enhances SOX2-mediated pluripotency during deprogramming.^83^ AKT phosphorylates SOX2 at T116, which protects SOX2 from ubiquitin-mediated degradation, thus contributing to tumorigenesis.^87^ SOX2 phosphorylation on T118 residue by PKCβ is associated with its transcriptional activity, as evidenced by the observation that the transcriptional activity is detected in either wild-type or SOX2 phospho-mimic mutant (SOX2-T118D), but not in SOX2 phospho-dead mutant (T118A).^88,89^ The SOX2 phosphorylation on the serine triplet S249-S250-S251 appears to repress SOX2 activity by regulating SUMOylation, another type of posttranslational modification.^90,91^ Human and mouse SOX2 are known to be SUMOylated on K245 or K247, respectively, which causes reduced SOX2 activity to trigger differentiation of ESCs.^84,90,91^ Notably, SUMOylation of SOX2 on K245 can be abolished in the SOX2 triplet mutant (S249A-S250A-S251A), suggesting that phosphorylation of the triplet serves as a priming step for subsequent SUMOylation of SOX2.^91^ A later study confirms that SOX2 phosphorylation on S251 site by ERK1/2 could promote SOX2 SUMOylation in nasopharyngeal carcinoma (NPC) cells.^92^ Pias2 was identified as the SUMO E3 ligase to promote SOX2 SUMOylation, which inhibits SOX2 transcriptional activity in ESCs.^90^ The SUMOylated SOX2 has impaired property in its DNA binding and is readily subjected to autophagic degradation, thus SOX2 SUMOylation results in reduced stability and activity of SOX2, thereby reducing the stemness of NPC.^84,92^ On the other hand, methylation on R113 by coactivator-associated arginine methyltransferase 1 increases SOX2 self-association and facilitates SOX2-mediated transactivation.^93^ SOX2 is also monomethylated on lysine 42 and lysine 119 (equivalent to Lys-117 in human SOX2) by methyltransferase SET7, which triggers the ubiquitin-dependent proteolysis of methylated SOX2, thus promoting differentiation of ESCs^94,95^ (Figs. 1 and 3).

SOX2 is also subjected to acetylation. One example is by the p300/CBP acetyl-transferase which acetylates SOX2 on K75 residue to alter its nuclear localization.^96^ Specifically, acetylation-resistant (SOX2-K75A) SOX2 is retained and stabilized in the nucleus with increased transcriptional activity, whereas acetylation-mimic mutant SOX2-K75Q is largely excluded from nucleus by linking to the nuclear export machinery.^96^ Significantly, SOX2 acetylation is not only important for ESC functions, but also promotes reprogramming of somatic cells to iPSCs, when being maintained at a low level by deacetylase Sirtuin 1.^96,97^ Furthermore, SOX2 is subjected to modification by poly(ADP-ribose) polymerase-1 (PARP1).^98^ The PARP1 imposed PARPylation of SOX2 is essential for dissociation of excessive SOX2 from the enhancer of *FGF4*, as such SOX2 plays an important role in regulation of ESCs differentiation.^98^ Moreover, SOX2 has been shown to be O-glycosylated by O-GlcNAc transferase (OGT) at residues of Ser246/248.^99,100^ The O-glycosylation of SOX2 inhibits the SOX2–PARP1 interaction and decreases reprogramming efficiency in murine ESCs and iPSCs.^99,100^ More importantly, OGT is significantly overexpressed in pancreatic cancer,^101^ and the O-glycosylation of SOX2 by OGT stabilizes SOX2, thus promoting the self-renewal of pancreatic cancer cells^102^ (Figs. 1 and 3).

Finally, the posttranslational modification that precisely controls the SOX2 protein levels is via ubiquitylation and subsequent degradation by proteasomal or autophagic pathways.^94,103^ Other types of SOX2 posttranslational modification either facilitate or inhibit SOX2 ubiquitylation. For example, EGFR-induced SOX2 phosphorylation on Y277 significantly suppresses SOX2 ubiquitylation, leading to SOX2 stabilization, which contributes to the stemness and progression of oral cancer,^104^ whereas the p300/CBP-mediated SOX2 acetylation on K75 promotes the polyubiquitination of SOX2 and proteasomal degradation in ESCs.^96^ The homologous to E6-AP C-terminus (HECT)-type E3 ligase WW domain-containing protein 2 (WWP2) is found to specifically interact with SOX2 through its HECT domain and promote SOX2 ubiquitylation and degradation, which is required for appropriate differentiation of ESCs in development.^94^ Interestingly, in human esophageal cancer cells, AKT was reported to phosphorylate SOX2 at T116, which blocks the interaction between E3 ligase UBR5 and SOX2 to protect SOX2 from UBR5-mediated ubiquitination and degradation, thus enhancing SOX2-mediated proliferation and stemness of cancer cells.^87^ Consistently, SOX2 monomethylation at K119 by SET7 is required for SOX2 recognition by WWP2 for degradation, whereas SOX2 phosphorylation at T118 (equivalence of human T116) by Akt1 blocks SOX2 monomethylation, leading to SOX2 stabilization for the maintenance of ESCs.^94^ Furthermore, SET7-mediated SOX2 methylation at K42 is preferably recognized by ubiquitin E3 ligase CRL4^DCAF5^ for subsequent SOX2 ubiquitylation and degradation, which is another mechanism of controlling the self-renewal and pluripotency of ESCs.^105^ On the other hand, protein demethylase LSD1 and the methyl-binding protein PHF20L1 are likely to prevent SET7-mediated SOX2 monomethylation to inhibit SOX2 ubiquitylation and degradation to maintain the self-renewal of ESCs.^95^ Ubiquitin-conjugating enzyme E2S (UBE2S) decorates SOX2 through the formation of K11-linked polyubiquitin chains at K123 to facilitate SOX2 proteasomal degradation, thus regulating the self-renewal and pluripotent status of ESCs and repressing SOX2-mediated ESC differentiation toward the neural ectodermal lineage.^106^ Finally, cullin-RING ligase CRL4A^DET1-COP1^ promotes SOX2 ubiquitylation on multiple lysine residues for proteasomal degradation, therefore enhancing neural progenitor cell differentiation, whereas deubiquitylase OTUD7B removes poly-Ub conjugates from SOX2 to maintain its stability.^107^ Notably, it was the first study to show that COP1, as a substrate receptor, directly interacts with SOX2 for its ubiquitylation by CRL4, whereas OTUD7B is the first identified deubiquitylase to remove ubiquitin conjugates from SOX2, leading to its stabilization. Thus COP1 and OTUD7B coordinately govern the maintenance and differentiation of neural progenitor cells by modulating SOX2 stability.^107^ A subsequent study confirms the functional role of COP1 in the developing mouse brain.^108^ Taken together, SOX2 is subjected to precise regulations at the multiple levels by a variety of mechanisms to ensure its proper level and activity during normal development and physiological processes (Figs. 1 and 3).

### SOX2 cross-talks with multiple signaling pathways

#### The EGFR/MAPK/PI3K-mTORC-AKT signaling pathways

EGFR is a family member of receptor tyrosine kinases expressed on the cell membrane that binds to its ligands (members of the EGF family of proteins) to produce mitogenic effects in target cells, thus participating in various vital physiological processes, such as cell proliferation, differentiation, migration, and survival.^109^ Inhibition of EGFR signaling, through pharmacological inhibition or genetic inactivation, significantly reduces the SOX2 expression and subsequently suppresses the self-renewal of lung cancer stem-like cells.^110^ On the other hand, ectopic expression of a constitutively active EGFR mutant or ligand exposure induces SOX2 accumulation to further enhance the EGFR-dependent self-renewal capacity of lung cancer cells.^111^ Interestingly, SOX2 directly binds to the *EGFR* promoter region at 389–383 bp upstream of its transcriptional start site to enhance *EGFR* transcriptional expression, thereby promoting oncogenic phenotypes of lung cancer cells.^111^ The downstream signaling pathways of EGFR, including the mitogen-activated protein kinase (MAPK) and the phosphoinositide-3-kinase (PI3K)/AKT/mTOR are initiated upon the ligand–receptor binding.^112^ Activation of the MAPK signaling, through conditional expression of the gain-of-function alleles *BrafV600E*, induces *SOX2* transcription, which contributes to the pathogenesis of craniopharyngioma.^113^ In skin keratinocytes, SOX2 overexpression promotes cell proliferation and migration via activating the EGFR/MEK/ERK pathway to accelerate cutaneous wound healing.^114^ In hepatoma cells, the PI3K/AKT/mTOR signaling mediates SOX2 induction by cyclin G1 to promote the self-renewal, chemoresistance, and tumorigenicity.^115^ On the other hand, SOX2 inhibits mTOR via transcriptional repression to induce macroautophagy, which is required for nuclear reprogramming and iPSC formation.^116^ Moreover, AKT directly phosphorylates mouse SOX2 on T118, which stabilizes the SOX2 in ESCs.^94^ Conversely, inhibition of PI3K/AKT directly by PI3K inhibitor duvelisib promotes CSCs differentiation by reducing SOX2.^117^ Finally, during the progression of premalignant squamous lesions in tracheobronchial basal cells, SOX2 amplification cooperates with the PI3K/AKT signaling to promote squamous metaplasia at the expense of normal mucociliary differentiation^118^ (Fig. 4).

#### The SHH signaling pathway

Sonic hedgehog (SHH) is a secreted signaling protein that plays many important roles in cerebellar development and CNS development.^119^ SOX2 is specifically expressed in SHH-associated medulloblastoma, and exogenous administration of Shh significantly increases SOX2 expression in cerebellar granule neuron precursors and promotes cell proliferation.^120^ Moreover, the downstream factors of SHH signaling, GLI1/2 positively regulates *SOX2* expression by binding to its proximal promoter, which contributes to the self-renewal and tumorigenesis of melanoma.^121^ Conversely, Shh itself is a SOX2 transcriptional target in neural stem cells. This Shh regulation by SOX2 is important for neural stem cell maintenance and hippocampal development. Indeed, *SOX2* deletion in adult mice causes hippocampal neurogenesis defect, a phenotype also seen upon *Shh* loss.^119^ Furthermore, activation of the Shh signaling pathway by Shh pharmacological agonist remarkably rescues the hippocampal neurogenesis induced by SOX2 knockdown, suggesting a causal role of the SOX2–Shh axis in hippocampal development^119^ (Fig. 4).

#### The Hippo signaling pathway

The Hippo pathway is an evolutionarily conserved signaling cascade that controls the growth of cell, tissue, and organ in response to diverse environmental cues.^122^ During the airway development, Hippo pathway effector Yap determines the fate of epithelial progenitor cells and controls morphogenesis by cooperating with SOX2. When epithelial tubules start to form and branch, a nucleocytoplasmic shift of Yap is initiated to mark the boundary between the distal (SOX9-expressing) and the airway (SOX2-expressing) compartments. Nuclear Yap specifies a unique transcriptional program at the transition zone that regulates SOX2 expression for specification of airway epithelial cell precursors.^123^ Conversely, in the absence of Yap, epithelial progenitors fail to form normal airways because of uncontrolled distribution of SOX2 levels.^123^ Interestingly, SOX2 also directly modulates the Hippo pathway. SOX2 on one hand, directly transactivates YAP expression to antagonize the Wnt/β-catenin signaling to promote adipogenesis,^124^ and on the other hand, blocks the Hippo pathway by directly repressing the transcription of the Hippo activators, NF2 (Merlin) and WWCI (Kibra), leading to enhanced YAP function.^125^ YAP depletion causes reduction of CSCs and tumorigenicity of osteosarcomas. Thus, SOX2 interferes with the tumor-suppressive Hippo pathway to maintain CSCs in osteosarcomas^125^ (Fig. 4).

#### The Wnt/β-catenin signaling pathway

The Wnt/β-catenin signaling pathway plays an essential role in development and tumorigenesis.^126^ Several lines of evidence have showed that SOX2 cooperates with Wnt/β-catenin signal in dictation of cell lineages during proper tissue development. For instance, SOX2 activity is essential for mouse tooth development, and temporal knockdown of *SOX2* terminates the migration of dental epithelium cells, which is attributed to the degradation of Wnt/β-catenin signaling.^20,127^ The analysis of deletion or chimeric fusion mutants showed that SOX2 is capable of binding to *β-catenin* via its C-terminal transactivation region to interfere the Wnt signaling pathway, and by doing so SOX2 maintains the self-renewal capacity of osteoblast lineage.^128^ SOX2 depletion in murine osteosarcoma-derived cells accelerates the differentiation into mature bone-forming cells, and dramatically impairs their ability to form tumors due to inactivation of Wnt/β-catenin signaling. Conversely, activation of Wnt/β-catenin signaling suppresses the SOX2 expression and maintains osteosarcoma cells in a differentiated osteoblast-like state.^129^ During retina development, SOX2, on one hand, antagonizes the Wnt/β-catenin pathway to maintain the optic cup a neurogenic fate, and on the other hand, regulates cycling of optic cup progenitors in a Wnt-independent manner.^130^ In addition to directly binding to *β-catenin*, SOX2 also induces the expression of many Wnt signaling inhibitors, such as dickkopf-1, adenomatous polyposis coli, and GSK3β, to antagonize the Wnt/β-catenin signaling.^128,129^ In some cases, however, SOX2 could directly activate *β-catenin* expression by binding to its promoter, and also induces translocation of β-catenin from the cytosol to the nuclei, therefore activating the Wnt pathway to promote tumor metastasis.^131,132^ Thus, SOX2 plays a context-dependent role when cross-talking with Wnt signals (Fig. 4).

#### The TGF-β/Smad signaling pathway

Transforming growth factor (TGF-β)/Smad signaling is another important regulatory cascade for cell proliferation, differentiation, developmental patterning, morphogenesis, and disease pathogenesis.^133^ During the process of iPSCs generation, inhibition of TGF-β signaling appears to increase the reprogramming efficiency via bypassing the requirement for exogenous SOX2 expression.^134^ In glioma-initiating cells, TGF-β signaling induces SOX2 expression to maintain cell stemness, whereas inhibition of TGF-β signal deprives tumor cells of stemness and promotes cell differentiation via decreasing SOX2 expression.^41^ In melanoma, TGF-β causes SOX2 accumulation, while SOX2 depletion attenuates TGF-β-induced tumor invasiveness.^135^ In addition, SOX2 overexpression facilitates TGF-β-induced epithelial to mesenchymal transition (EMT) process, thus promoting lung cancer cell migration and invasion^136^ (Fig. 4).

## Role in regulation of tumorigenesis and drug resistance

### Role in the maintenance of cancer stemness

CSCs are a small population of primary tumor cells that are characterized by self-renew capacity, higher tumorigenesis, and resistance to therapies.^137^ CSCs are therefore considered as a main cause of cancer recurrence, distant metastasis, and drug resistance.^138^ As an important transcription factor contributing to the stemness of pluripotent stem cells, SOX2 also plays a critical role in regulation of self-renewal and stemness of CSCs.^58,139,140^ SOX2 overexpression links to cancer-stem-cell characteristics of colorectal cancer,^141^ and the elevated level of SOX2 was found in osteosarcoma stem cells isolated by a sphere-forming assay.^142^ Ectopic SOX2 expression is also significantly associated with increased population of CSCs in lung and ovarian cancers,^143,144^ whereas SOX2 knockdown dramatically reduces the percentage of CSCs and inhibits the formation of xenograft tumors.^121,145^ Likewise, SOX2 inhibition by a miRNA decreases the breast CSC population and attenuates its tumorigenicity in NOD/SCID nude mice.^146^ Interestingly, when breast cancer cells are co-cultured with the immature adipocytes, the number of breast CSCs is significantly increased through SOX2 activation.^147^ In pancreatic cancers, SOX2 expression is frequently and specifically elevated in CSC populations, accompanying with an increased expression of other pancreatic CSC markers, including ALDH1, ESA, and CD44.^148^ Although growing pieces of evidence have demonstrated that SOX2 promotes the CSCs maintenance in different types of human cancers,^41,149–151^ it is worth noting that SOX2 is expressed heterogeneously in various types of cells throughout tumor tissues, and indeed, only a small population of cells is SOX2 positive in some tumors.^149,152^ Thus, the conclusion on the causal role of SOX2 in CSCs should be derived from the studies using isolated SOX2-positive subpopulation of tumor cells, rather than from the overall elevated SOX2 levels in the entire tumor tissues. Notably, the tracing experiments by the knock-in of GFP-encoding gene into the endogenous *SOX2* loci demonstrated that the SOX2-positive green cells isolated from heterogeneous tumor cell populations indeed express higher levels of stemness-related genes with characteristics of stem cells, as compared with SOX2-negative counterparts,^149,153^ confirming a causal role of SOX2 in cancer cell stemness.

### Role in growth and survival of cancer cells—cell culture models

**Fig. 5:**
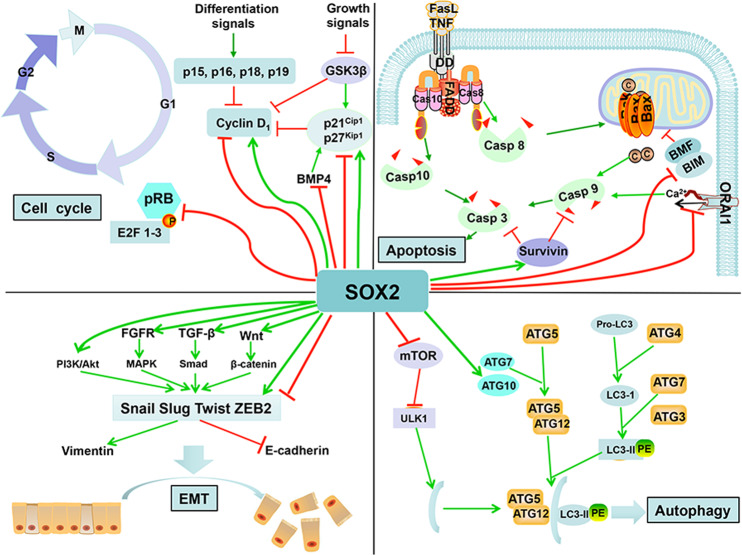
SOX2 regulates a variety of biological processes. SOX2 regulates various biological processes, leading to oncogenic consequence. In general, SOX2 represses cell cycle upon differentiation signals to maintain the stemness and accelerates cell-cycle progression upon growth signals for proliferation. SOX2 exerts an antiapoptotic effect to promote survival of cancer cells. SOX2 also triggers EMT process to enhance tumor invasion and metastasis. Finally, SOX2 induces cancer-specific autophagy to confer chemoresistance

Accumulated lines of evidence strongly indicate that SOX2 plays a tumor promoting role in human cancers. Dysregulation of SOX2 has been found in over 20 different types of human cancers,^5^ and SOX2 overexpression and associated abnormal cross-talks with multiple signaling pathways trigger several malignant features, including uncontrolled proliferation, resistance to apoptosis, altered autophagy, enhanced EMT, and maintenance of CSCs, leading to tumor progression, metastasis, and drug resistance (Figs. 4 and 5). Thus, SOX2 is indeed an attractive cancer target.^7,154^

#### Cell proliferation

SOX2 expression in stem cells maintains a cell self-renewing status by repressing the expression of several proliferative genes, whereas SOX2 depletion promotes cell proliferation by derepression of cyclin D1 to drive cells toward a differentiation state.^155^ Indeed, SOX2 expression in cancer cells drives cell proliferation. For example, SOX2 facilitates pancreatic cancer cell-cycle progression through activating cyclin D3 and downregulating p21^Cip1^ and p27^Kip1^ in a cell-cycle-dependent manner.^148^ Likewise, SOX2 knockdown in prostate cancer cells causes downregulation of cyclin E and upregulation of p27, thereby inhibiting G1 to S transition, while SOX2 overexpression causes the opposite effect.^156^ Furthermore, SOX2 could promote cell proliferation in a manner independent of cell cycle by activating AKT/mTORC1 pathway in esophageal squamous cell carcinoma (ESCC), where *SOX2* gene is frequently amplified.^157^ SOX2 also represses the expression of antiproliferative factor BMP4 by directly binding to its first intron region, therefore promoting cell-cycle progression of lung squamous cell carcinoma (LSCC).^158^ Moreover, SOX2 expression is also required for cell proliferation of laryngeal and ESCCs,^159^ osteosarcoma,^160^ breast cancer,^68^ and ovarian cancer.^161^ In contrast, some studies controversially show that SOX2 causes cell-cycle arrest at the G0/G1 phase and suppresses cell proliferation via the mechanisms involving cyclin D1 downregulation, Rb phosphorylation, p27^Kip1^ increase, and inactivation of mTOR pathway in colorectal and GC cells.^45,162^ Thus, SOX2 effects on cell-cycle progression could be context-dependent. In general, SOX2 blocks cell cycle upon differentiation signals to maintain the stemness and accelerates cell cycle upon growth signals for cancer cell proliferation (Fig. 5).

#### Apoptosis

As an oncoprotein, SOX2 expression confers cancer cells resistance to apoptosis. Inhibition of SOX2 significantly induces apoptosis, and suppresses the metastatic potential in syngeneic mouse models of breast and lung cancers.^111,163,164^ One mechanism is through upregulation of an apoptotic inhibitor, survivin. SOX2 knockdown reduces survivin expression, thus activating the caspase-9-related mitochondrial apoptotic pathway to induce apoptosis in neural stem cells,^165^ and lung cancer cells,^166^ which can be largely rescued by survivin overexpression, indicating a causal role of SOX2 in apoptosis protection.^166^ In Ewing’s sarcomas, SOX2 depletion apparently induces apoptosis by activating key proteins controlling both extrinsic death receptor pathways (caspases-8 and Fas) and intrinsic mitochondrial pathways (caspase-9 and Bad), highly suggesting that SOX2 inhibition could activate both apoptotic pathways.^167^ Furthermore, the BH3-only proapoptotic genes *BIM* and *BMF* were identified as SOX2 target genes by Chip-seq analysis. SOX2 binds to the transcriptional start sites of both *BIM* and *BMF* genes to repress their transcription, therefore inhibiting apoptosis in lung cancer cells following oncogene withdrawal.^168^ The perturbed intracellular Ca^2+^ signal and the store-operated Ca^2+^ entry (SOCE) activity are known to link to the antiapoptotic property in prostate cancer.^169^ Interestingly, SOX2 inhibits the expression of calcium release-activated calcium channel protein 1 (Orai1), therefore impairing the SOCE activity and alter Ca^2+^ homeostasis, eventually leading to apoptosis resistance in human prostate cancer cells.^170^ Finally, SOX2 inhibition also induces apoptosis in melanoma cells,^121^ triple negative breast cancer cells,^163^ and laryngeal squamous cell carcinoma cells.^171^ In general, SOX2 has the antiapoptotic effect in cancer cells (Fig. 5).

#### EMT

EMT is a process in which epithelial cells lose their intracellular adhesion ability, but acquire cell motility, thereby playing an important role in tumor invasion and metastasis.^172^ Numerous studies show that SOX2 promotes the EMT via multiple mechanisms. In non-small-cell lung cancer cells, SOX2 enhances the TGF-β-induced EMT process by negatively regulating TIF1γ expression.^136^ In breast and prostate cancer cells, SOX2 promotes EMT through the Wnt/β-catenin signaling pathway by directly binding to the *β-catenin* enhancer, therefore promoting tumor cell migration and invasion both in vitro and in vivo.^131^ In bladder cancer^173^ and lung cancer cells,^174^ SOX2 is required to act as an important bridge for FGFR/MAPK-induced EMT. Moreover, SOX2 induces phosphorylation of AKT and mTOR to increase expression and activity of matrix metalloproteinase-2, thus enhancing the EMT process in lung cancer cells.^175^ In ESCC, SOX2 activates the STAT3/HIF-1α signaling to upregulate Slug expression, thus promoting EMT process.^176^ SOX2 also activates the transcription of mesenchymal genes, such as *Snail*, *Slug*, and *Twist*, and downregulates the epithelial genes, such as *E-cadherin* and *ZO-1*, eventually driving EMT-induced tumor invasion in breast cancer and pancreatic cancer cells.^67,148,177^ Interestingly, in bladder cancer cells, TGFβ1, a potent EMT inducer, reduces the expression of the epithelial marker E-cadherin and increases expression of both SOX2 and NANOG to promote cell stemness, suggesting that EMT process is not only associated with tumor invasion, but also links to the initiation of SOX2-mediated stemness, and gain of epithelial stem cell properties.^178,179^ However, in some breast cancer cell lines, SOX2 overexpression impairs EMT process by downregulating transcription factor Twist1 via binding to an alternative SOX2 binding motif present in the *Twist1* gene promoter, therefore suppressing tumor invasion.^180^ Taken together, SOX2 mainly promotes the EMT, but the effect could also be cell context-dependent under certain circumstances.

#### Autophagy

Autophagy controls the intracellular protein quality of different systems and maintains homeostasis and cellular viability,^181,182^ and is critical for the maintenance of ESCs and CSCs by maintaining optimal levels of pluripotency transcription factors, such as OCT4, SOX2, and NANOG.^103,183^ The temporal regulation of autophagy by SOX2 is a critical step for cellular reprograming.^116^ The osimertinib-resistant lung cancer cells express high levels of SOX2 with increased autophagy and stem cell-like properties, and autophagy inhibitors effectively decrease SOX2 expression and cell stemness.^184^ SOX2 knockdown in lung cancer cells induces autophagy by enhancing the expression of autophagy marker LC3-II.^111^ Moreover, SOX2 induces autophagy by transcriptional repression of mTOR through binding and recruiting the nucleosome remodeling and deacetylase complex to its repressive region of promoter, which is an important step in reprogramming to pluripotency.^116,183^ In colon cancer cells, SOX2 induces cancer cell-specific autophagy by transactivating ATG10 expression, leading to growth suppression both in vitro and in vivo.^185^ In breast cancer, autophagy is required for the tumorigenicity of cancer stem-like/progenitor cells.^186,187^ Collectively, SOX2 regulates autophagy and is being regulated by autophagy as well in multiple human cancer cells. The autophagy inhibitors may have a nonspecific role in SOX2 targeting (Fig. 5).

### Role in tumorigenesis—genetically modified mouse models

SOX2 is an important transcriptional factor required for mouse embryonic development and tissue homeostasis, as revealed by total and conditional SOX2 knockout studies.^3,188^ The studies to determine the role of SOX2 in tumorigenesis, using genetically engineered mouse models, were mainly conducted under SOX2 transgenic expression or deletion alone or in combination with oncogene activation or tumor-suppressor inactivation (Table 1). For example, transgenic overexpression of homozygous *SOX2* in lung Clara cells induces hyperplasia of bronchial epithelial cells, eventually leading to lung adenocarcinoma in 50% of mice, implying the oncogenic role of SOX2 during lung tumorigenesis.^189^*SOX2* transgenic expression in combination of deletion of three tumor suppressors, *TP53*, *Pten*, and *Cdkn2A/p16* promotes the formation of LSCCs.^190^ Similarly, *SOX2* transgenic expression in combination of *Lkb1* deletion leads to mTORC pathway activation to promote LSCC tumorigenesis, and SOX2 also promotes recruitment of tumor-associated neutrophils.^191^ Moreover, *SOX2* overexpression with the loss of *Cdkn2ab* and *Pten* in tracheobronchial basal cells promotes the development of heterogeneous lesions to LSCCs.^192^ Likewise, *SOX2* overexpression with *Cdkn2ab* and *Pten* loss also drives alveolar type 2 (AT2) and club cells toward LSCCs,^192^ suggesting that SOX2 determines tumor lineage to LSCC independent of cells of origins.^193^ Interestingly, in the Kras^G12D^ mouse lung cancer model, homozygous *SOX2* deletion in CC10-positive AT2 cells caused early death, whereas heterozygous *SOX2* deletion leads to development of airway papillary adenocarcinoma at 12 weeks after Kras activation and SOX2 reduction. On the other hand, SOX2 overexpression suppresses tumor formation in K-Ras^G12D^-expressing AT2 cells. Thus, SOX2 appears to be a tumor suppressor in this model.^194^ In ESCC model, Krt-5-driven transgenic *SOX2* expression in basal progenitor cells triggers hyperplasia in esophagus and squamous cell carcinomas in the forestomach, particularly when cooperated with inflammation-mediated Stat3 activation in the latter case.^195^ In a genetically engineered mouse model of osteosarcoma, induced by dual deletion of *RB* and *p53* in the mouse osteoblast lineage, *SOX2* deletion dramatically delays and reduces the tumor formation, along with an extended lifespan. Interestingly, all the tumors derived from the *SOX2* knockout animals are stained positively with SOX2, suggesting that SOX2 is essential for tumorigenesis of osteosarcoma.^160^ Transgenic expression of *SOX2* in the external granule cells exhibits more proliferate potential, but gives rise to medulloblastoma when Shh signaling is being constitutively active.^196,197^ Consistently, *SOX2* deficiency suppresses the Shh signaling-induced tumor formation, implying that SOX2 expression in granule cell precursors is required for Shh-induced medulloblastoma^120^ (Table 1). Taken together, SOX2 is, in general, an oncogene that promotes tumorigenesis, particularly in combination with simultaneous activation or inactivation of dominant oncogenes or tumor suppressors, respectively.

**Table 1:** SOX2 regulates tumorigenesis in mouse models

GEMM	Genetic alteration	Phenotype	Reference
*Scgb1a1-Cre-ER* *Rosa26R-SOX2/+*	Inducible SOX2 overexpression in adult lung epithelial cells	Bronchial hyperplasia at 6 weeks; about half of mice developed adenocarcinoma at 18–40 weeks	^189^
*Ad5-K5-Cre or Ad5-K14-Cre* *LSL-* *SOX2* *Pten*^*fl/fl*^ *Cdkn2ab*^*fl/fl*^	SOX2 overexpression in tracheobronchial basal cells combined with Cdkn2ab and Pten loss	27% of mice showed lesions of atypical hyperplasia commonly in bronchi; 73% of mice developed multiple LSCC	^192^
*Ad5-SPC-Cre or Ad5-CC10-Cre* *LSL-* *SOX2* *Pten*^*fl/fl*^, *Cdkn2ab*^*fl/fl*^	SOX2 overexpression in alveolar type 2 (AT2) and club cells combined with Cdkn2ab and Pten loss	All mice developed peripheral tumors around 7–8 months after Cre activation, and more than 90% of cases were well to moderately differentiated LSCC	^192^
*KRT5-Cre-ER* *Rosa26R-SOX2/* *SOX2*	Inducible SOX2 overexpression in squamous epithelium	Basal cell hyperplasia in esophagus, SCC in forestomach by 13 weeks of age	^195^
*OSX-Cre* *Rb*^*fl/fl*^ *p53*^*fl/fl*^ *Sox2*^*fl/fl*^	SOX2 deleted in a p53 null, RB null, OSX-Cre expressing background	Osteoblast-specific Sox2 conditional knockout causes a drastic reduction in the frequency and onset of tumors	^160^
Intranasal inhalation *Ad5-CMV-CRE* *Rosa26R-SOX2/* *SOX2* *Lkb*^*fl/fl*^	SOX2 overexpression in lung combined with *Lkb* loss	Mice developed LSCC after an average of 11 months	^191^
*Ad5-CC10-Cre-ER* *Sox2*^*fl/+*^*LSL-K-Ras*^*G12D*^	SOX2 heterozygous deletion in alveolar type 2 (AT2) cells with *K-Ras*^*G12D*^	Mice developed airway papillary adenocarcinomas at 12 weeks after tamoxifen	^194^
*Ad5-CC10-Cre-ER* *LSL-* *SOX2* *LSL-K-Ras*^*G12D*^	SOX2 overexpression in AT2 cells with K-Ras^G12D^	Mice have markedly smaller alveolar tumors than CC10-CreER, K-Ras mice at similar ages	^194^

*GEMM* genetically engineered mouse model, *LSCC* lung squamous cell carcinoma

### Overexpression in human cancers and serves as a prognostic biomarker

**Fig. 6:**
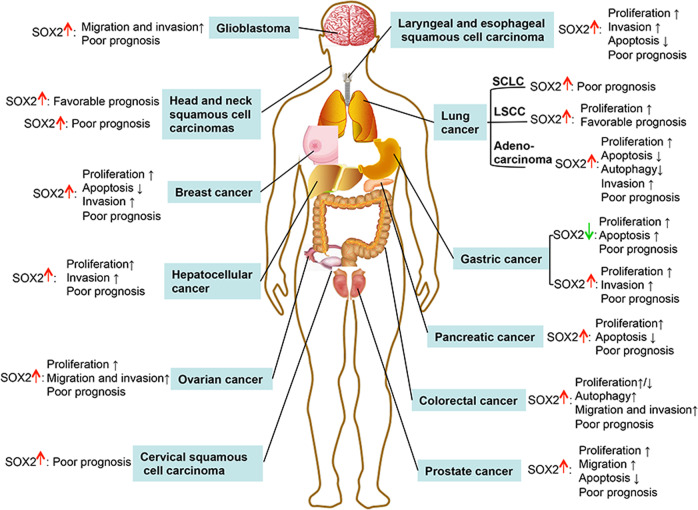
Correlation of SOX2 levels with prognosis of cancer patients. In most human cancers, SOX2 overexpression or amplification is associated with poor survival of cancer patients. In certain types of cancers, such as gastric cancer, head and neck squamous cell carcinoma, and LSCC, higher SOX2 levels are associated with a better patient survival. SCLC small cell lung cancer, LSCC lung squamous cell carcinoma

**Fig. 7:**
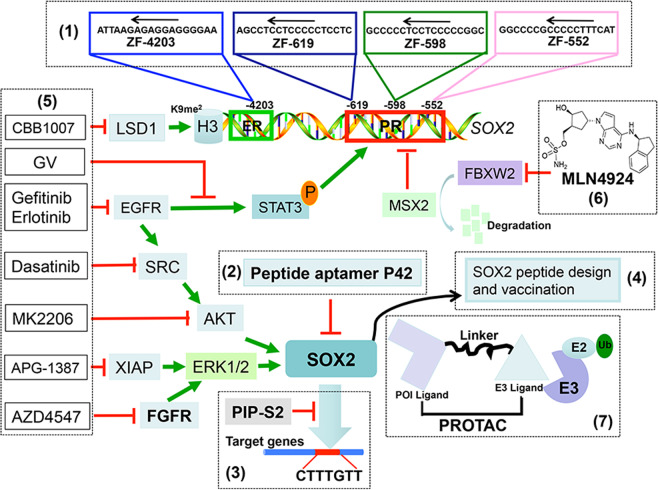
SOX2-targeting approaches. Currently, the approaches undertaken to target SOX2 includes: (1) to alter the endogenous SOX2 gene expression by direct gene targeting using the zinc-finger (ZF)-based artificial transcription factor (ATF); (2) to generate the peptide aptamer for SOX2 targeting; (3) to target the SOX2-DNA binding, thus inhibiting SOX2 transcriptional activity; (4) to target SOX2 via inducing immune responses; (4) to use small molecules inhibitors of signaling pathways that affect SOX2, thus indirectly inhibiting SOX2; (6) to target protein ubiquitylation and degradation to specifically shut down SOX2 expression; (7) to develop the PROteolysis Targeting Chimera (PROTAC) method to directly degrade SOX2

Given the critical role of SOX2 in the initiation and progression of tumorigenesis, it is not surprising that SOX2 dysregulation is frequently occurring in human cancer tissues. Indeed, SOX2 overexpression or amplification is often observed in many different types of human cancers, which, importantly, is correlated with poor survival of cancer patients (Fig. 6). In ovarian cancer, SOX2 expression is progressively increased from normal ovary tissues to ovarian cancer tissues,^198,199^ then to metastatic ovarian cancer tissues.^161^ And elevated SOX2 is correlated with worse prognosis of ovarian cancer patients.^144,200^ During lung tumorigenesis, *SOX2* amplification is more frequently detected in high-grade bronchial lesions than in low-grade lesions, with the former having a high frequency to progression into cancers.^201^ The *SOX2* amplification is also seen in lung adenocarcinomas,^202^ and serves as an independent poor prognostic marker.^203^ Immunohistochemical staining for SOX2 protein level in over 400 lung cancer tissues revealed that higher SOX2 staining is in both small cell lung cancer (SCLC) and non-SCLC tissues than in normal tissues,^204^ and elevated SOX2 is significantly associated with high-grade histology and poor prognosis of SCLCs.^205,206^ Interestingly, SOX2 mRNA is detectable in the sera from SCLC patients,^207^ and the high SOX2 mRNA levels in blood samples of SCLC patients predict poor progression-free survival and overall survival.^208^ Furthermore, *SOX2* amplification occurs frequently in the majority of LSCCs,^151,209,210^ but the elevated SOX2 levels unexpectedly predict a favorable prognosis for LSCC patients.^211,212^ A logical explanation is that SOX2 harbors an oncogenic potential during LSCC tumorigenesis, but the tumors arising from SOX2 overexpression exhibits a clear squamous cell differentiation, therefore conferring better prognostic features.^212^ In glioblastoma multiforme (GBMs), SOX2 overexpression at both mRNA and protein levels are frequently occurring and positively associated with malignancy grades.^213^ The invasive areas of GBMs, composing of a large number of dedifferentiated tumor cells,^214,215^ are highly SOX2-positive staining, which is considered as a biomarker of tumor aggressiveness and poor outcomes.^216^ Targeting SOX2 is, therefore, considered as an effective therapeutic approach in the treatment of GBMs.^217,218^ In colorectal cancers, SOX2 expression is associated with cancer stem-like properties, correlates with lymph-node metastases and distant spread, and poor patient prognosis.^219–221^ The immunohistochemical staining of SOX2 in various stages of prostate tumorigenesis from benign prostate hyperplasia, primary prostate cancer to metastatic prostate cancer tissues revealed that SOX2 is mainly expressed at the metastatic sites.^222^ In prostate cancers, elevated SOX2 levels are significantly associated with lymphovascular invasion and shorter latent period of disease recurrence.^223,224^ Likewise, SOX2 is significantly upregulated in highly metastatic liver cancer cells, as compared with the low metastatic counterparts, and elevated SOX2 expression predicts tumor metastasis and poor patient survival.^225^ Moreover, SOX2 overexpression is positively associated with worse clinicopathological parameters and poor prognosis of patients with head and neck squamous cell carcinomas (HNSCCs),^226^ upper urinary tract urothelial cell carcinomas,^227^ laryngeal squamous cell carcinomas,^228^ pancreatic cancers,^229^ cervical squamous cell carcinomas,^230^ and breast cancers.^231–234^ The roles of SOX2 in HNSCCs and GCs are rather controversial.^226,235,236^ Specifically, in HNSCC, SOX2 overexpression is frequently detected in primary tumors but not in healthy tissues, and elevated expression of SOX2 is significantly correlated with cancer recurrence and poor prognosis.^145,226^ However, the subsequent study showed that patients with SOX2^high^ HNSCCs have markedly longer progression-free survival (51 vs. 16 months) and overall survival (110 vs. 37 months) than those with SOX2^low^ tumors.^236^ In GCs, some studies showed that SOX2 is downregulated in both cancer cell lines and tissues,^45,237^ and elevated SOX2 expression is correlated to lower tumor stage, lower incidences of lymph-node metastasis, and better prognosis,^45^ whereas other studies supported that SOX2 exerts oncogenic effect during gastric tumorigenesis, given that SOX2 overexpression is related to tumor invasion, lymph-node metastasis, and worse outcome.^238,239^ Overall, SOX2 amplification or overexpression is frequently observed in most human cancers, which is highly associated with poor survival of cancer patients. Thus, SOX2 is, in general, an oncogenic protein to promote human tumorigenesis, and a validated cancer drug target (Figs. 6 and 7).

### Role in drug resistance

The development of drug resistance in cancers is closely related to worse clinical prognosis and represents a huge challenge that drastically limits the efficacy of the current anticancer therapies. Accumulated data have demonstrated that SOX2 upregulation in cancer cells is frequently linked to resistance to anticancer drugs.^33,240^ There is a plethora of different mechanisms contributing to SOX2-induced therapy resistance. One important mechanism is that SOX2 expression in cancer cells is associated with a CSC state, which is defined as a subpopulation cells within the tumor that are shown to be more resistant toward cancer therapies.^154,241,242^ For example, the tamoxifen-resistant breast cancer cells exhibit stem cell-like features with a high SOX2 level, and SOX2 knockdown restores the sensitivity toward tamoxifen, whereas SOX2 overexpression confers tamoxifen resistance.^243^ Importantly, our recent study showed that MLN4924, a small molecular inhibitor of protein neddylation,^51^ sensitizes otherwise resistant breast cancer cells to tamoxifen by downregulating SOX2 through inactivation of FBXW2 E3 ligase.^7,52^ One study showed that tumor-associated macrophages are able to maintain stem cell phenotype of breast cancer by inducing SOX2 expression, eventually contributing to mitoxantrone resistance.^244^ Similarly, SOX2 phosphorylation mediated by activated PI3K/AKT prevents ubiquitin-mediated degradation of SOX2 and causes SOX2 accumulation, which is associated with drug resistance to R-CHOP (rituximab/R, cyclophosphamide/C, doxorubicin/H, vincristine/O, and prednisone/P) in diffuse large B-cell lymphoma, along with increased CSC proportion.^117^ In addition, SOX2-induced protective autophagy also links to drug resistance. For instance, the osimertinib-resistant lung cancer cells express higher SOX2 levels with higher autophagic flux levels, and SOX2 knockdown or autophagy inhibitor treatment resensitizes these resistant cells to osimertinib.^184^ Moreover, SOX2 overexpression renders ovarian cancer cell resistance to many chemotherapeutic drugs, such as carboplatin, cisplatin, and paclitaxel, by efficiently repressing apoptosis via inducing antiapoptotic proteins and inhibiting proapoptotic proteins.^144^ Consistently, SOX2 knockdown confers susceptibility of cancer cells to paclitaxel and cisplatin by inducing autophagy, apoptosis, and mitochondrial abnormalities.^111,245^ In SCLCs, SOX2 confers melanoma cell adhesion molecule (MCAM)- induced chemoresistance to doxorubicin, cisplatin, or etoposide by increasing the expression of mitochondrial 37S ribosomal protein 1/ATP-binding cassette subfamily C member 1, and activating the PI3K/AKT pathway.^246^ In EGFR-mutated lung adenocarcinoma cells, SOX2 is markedly induced after treatment with erlotinib, which represses the expression of proapoptotic proteins, BIM and BMF, thus contributing to erlotinib resistance.^168,247^ Moreover, SOX2 overexpression in *TP53*- and *RB1*-deficient prostate cancer cells is sufficient to trigger resistance to the antiandrogen drugs, along with a phenotypic shifting from androgen receptor (AR)-dependent to AR-independent.^248^ Ectopic overexpression of SOX2 is also linked to paclitaxel resistance by inducing PI3K/AKT pathway in prostate cancer cells.^249^ In GC stem-like cells, SOX2 knockdown improves the sensitivity of doxorubicin by inhibiting drug efflux.^250^ In melanoma cells, SOX2 expression induced upon BRAF inhibitor treatment leads to increased transcription of CD24 to activate the SRC and STAT3 signaling, thus causing resistance to BRAF inhibitor.^251^ In gliomas, SOX2 expression is associated with temozolomide resistance via activation of the mTOR pathway,^252^ and resistance to 1,3-bis(2-chloroethyl)- 1-nitrosourea via upregulation of the ABCC3 and ABCC6 transporters.^58^ In melanoma, SOX2 transactivates the ABCC1 by binding to *ABCC1* promoter, which contributes to paclitaxel resistance.^253^ In head and neck SCCs, SOX2 triggers cisplatin resistance by inducing ABCG2 transporter expression, whereas SOX2 knockdown reestablishes the sensitivity to cisplatin, indicating a causal role of SOX2.^145^ Taken together, SOX2 actively confers resistance of various types of cancer cells to chemotherapeutic drugs. SOX2 is, therefore, an attractive therapeutic target for overcoming drug resistance.

## SOX2-targeting approaches

For most human cancers, SOX2 acts as an oncoprotein by activating several proliferative and antiapoptotic signal cascades to promote tumorigenesis, metastasis, and drug resistance. Targeting SOX2 is, therefore, an effective strategy for anticancer therapy. However, given the nature that SOX2 is a undruggable transcription factor, the progress in the discovery of selective SOX2 inhibitors is very limited, although several SOX2-targeting approaches have been undertaken (Fig. 7).

The first approach is to alter the endogenous *SOX2* gene expression by direct gene targeting, using the zinc-finger (ZF)-based artificial transcription factor (ATF), which is designed to specifically bind to the genomic sequences of the gene of interest.^254^ Four distinct ATFs have been engineered with three (ZF-552SKD, ZF-598SKD, and ZF-619SKD) binding to the proximal *SOX2* promoter, and one (ZF-4203SKD) binding to the *SOX2* enhancer, *SRR1*. Among them, ZF-552SKD and ZF-598SKD are the most potent to reduce *SOX2* mRNA levels in MDA-MB-435s cells by 74% and 94%, respectively. The in vivo xenograft experiments verified that ZF-598SKD significantly inhibits tumor growth of breast cancers with a long-term effect.^255^ Likewise, ATF-based SOX2 inhibition technology also shows impressive effect in growth suppression of SCC in lung and esophageal cancers.^256^ However, ZF-ATFs in most cases are delivered by virus, limiting its utility due to poor delivery efficiency and nonspecific nature of viral infections.

The second approach is to generate the peptide aptamer for SOX2 targeting. The peptide aptamer P42 possesses a constrained peptide expression cassette in the active site loop of thioredoxin and a partial fragment of Venus protein, which is capable of efficiently interacting with SOX2 and inhibiting SOX2 downstream genes.^257^ It has been recently demonstrated that P42 can effectively reduce SOX2 function and apparently decrease the proliferation and migration of human esophageal cancer cells both in vitro and in vivo.^257,258^

The third approach is to target SOX2-DNA binding, thus inhibiting SOX2 transcriptional activity. PIP-S2, a hairpin pyrrole–imidazole polyamides (PIPs)-based bioactive synthetic DNA-binding inhibitor could inhibit the SOX2-DNA binding by competition with SOX2 for its consensus DNA-binding sequence (5′-CTTTGTT-3′), which in turn alters the SOX2-associated gene regulatory networks, leading to mesoderm differentiation from iPSCs.^259^ However, the therapeutic application for such approach is questionable.

The fourth approach is to target SOX2 via immunological response. One study used SOX2 as a glioma-specific antigen for T-cell immunotherapy of glioma patients, given that SOX2 is overexpressed in glioma cells and tumor tissues. Interestingly, SOX2-derived peptides can activate cytotoxic T lymphocytes to lyse glioma cells.^260^ Another study used SOX2 peptides for mouse immunization and found that the immunized mice display a delayed tumor onset and prolonged survival.^261^

The fifth approach is to use small molecules that target signaling pathways, which regulate SOX2 or being regulated by SOX2 to inhibit SOX2 indirectly. For examples, X-linked inhibitor of apoptosis proteins (XIAPs) was shown to protect SOX2 from autophagic degradation in NPC.^92^ An XIAP inhibitor (APG-1387) has an antitumor effect on CSCs expressing high level of SOX2 with a synergistic effect in combination with CDDP/5-FU.^92^ In LSCCs, the inhibitor of histone demethylase LSD1 (CBB1007) suppresses growth of SOX2-expressing cancer cells, since LSD1 likely serves as an epigenetic target in SOX2-expressing cancers.^262^ Furthermore, SOX2 is regulated by the EGFR–SRC–AKT and FGFR–ERK1/2 signaling, and inhibitors of their pathways would block self-renewal and expansion of stem-like cells by indirectly targeting SOX2.^110,174^ As such, EGFR inhibitors, Gefitinib and Erlotinib, Src inhibitor Dasatinib, AKT inhibitor MK2206, and FGFR1 inhibitor AZD4547 are all demonstrated to indirectly targeting SOX2, eventually suppressing the self-renewal properties and tumor growth of lung cancer^110,174^ and esophagus squamous carcinoma^87^ using in vivo mice models. Furthermore, in melanoma cells, triphenylmethane gentian violet (GV) inhibits survival and self-renewal capacity by blocking SOX2 induction via the EGFR–STAT3 signaling.^42^

The sixth approach is to target protein degradation to shut down SOX2 expression. We recently found that neddylation inhibitor MLN4924 effectively blocks SOX2 expression in many types of human cancer cells by targeting the FBXW2–MSX2–SOX2 axis. Mechanistically, SOX2 transcription repressor MSX2 is an ubiquitylation substrate of FBXW2 E3 ligase. By inactivating FBXW2 E3, MLN4924 causes accumulation of MSX2 to transcriptionally repress SOX2 expression.^52,263^ Biologically, MLN4924, via depleting SOX2, overcomes tamoxifen resistance of breast cancer cells, as demonstrated by both in vitro proliferation and survival assays, and in vivo xenograft tumor model^52^ (Fig. 7).

## Summary and future perspectives

SOX2 is a growth essential gene required for embryogenesis, organ development, and tissue homeostasis. The dynamic expression of SOX2 is strictly controlled by a complex regulatory network at the multiple levels, including transcription, post-transcription, and post-translation. SOX2 also cross-talks with multiple signaling pathways to ensure proper regulations of important biological processes, including cell-cycle progression, apoptosis, autophagy, and EMT. Aberrant expression of SOX2 affects self-renewal of ESCs and causes abnormal proliferation. The preclinical studies using both cell culture and genetically modified mouse models strongly suggest that SOX2 is an oncogenic protein. Furthermore, in most cancer types, SOX2 amplification or overexpression is a frequently occurring event, which is associated with advanced stages of tumorigenesis, poor prognosis, and drug resistance. Thus, SOX2 is well-validated cancer target. However, the underlying mechanisms of how SOX2 promotes tumorigenesis at each disease stage in a context-dependent manner, and why under certain circumstances, SOX2 acts as a tumor suppressor are interesting topics for future investigation.

The biggest future challenge with therapeutic application is to discover small molecule inhibitors, that directly target SOX2 effectively as an undruggable transcription factor, given ineffectiveness of current targeting approaches. The development of PROteolysis Targeting Chimera (PROTAC) provides such an opportunity. PROTAC is an emerging technique that links target-binding small molecule to cullin-RING ligase for proteasomal degradation of any given protein of interest.^264^ Specifically, PROTAC consists two functional parts, connected by a chemical linker: a “warhead” (normally a small molecule) that binds to a targeted protein, and a ligand which is recognized by a cullin-RING ligase, leading to degradation of the target protein.^264^ As such, PROTAC provides an effective approach to target these undruggable proteins,^265–267^ such as transcriptional factor SOX2 (Fig. 7, approach 7). At the present time, there are no small molecules known to bind to SOX2, unfortunately. Some peptide aptamers, such as P15, P18, and P24, have been identified to directly bind SOX2.^257^ Whether these synthetic aptamers (certainly not small molecules in nature) are useful as the “warhead” to target SOX2 via PROTAC strategy is an open question. Nevertheless, discovery and development of the PROTAC compounds to selectively target SOX2 would have unique applications for the treatment of human cancers with SOX2 overexpression. Given its fundamental role in embryonic development^3^ and epithelial tissue homeostasis,^188^ associated cytotoxicity of SOX2 targeting should be an important issue to consider. A newly developed light-inducible switch on PROTAC, which triggers the degradation of any given targeted proteins restrictedly at desirable site and time window by ultraviolet irradiation,^267,268^ should be incorporated in the SOX2-targeting strategy of PROTAC to overcome the potential side-effects.
